# Stabilized sand seas facing climate threats need health baselines now

**DOI:** 10.1016/j.eehl.2026.100272

**Published:** 2026-08-22

**Authors:** Shivukumar Rakkasagi, Dorit Nitzan, Shimrit Maman

**Affiliations:** a School of Public Health, Ben-Gurion University of the Negev, Be'er Sheva, Israel; b Homeland Security Institute, Ben-Gurion University of the Negev, Be'er Sheva, Israel; c Israel Space Agency, Ministry of Innovation, Science and Technology, Israel

Central Asia’s Karakum and Kyzylkum sand seas, covering about 500,000 km^2^, represent an unrecognized form of health infrastructure. Remote sensing reveals that the total desert land is nearly 95% stabilized by biogenic crusts and psammophylic plants [1,2], contradicting decades of desertification narratives. This stability is not merely ecological; it is a critical One Health anchor preventing catastrophic dust exposure for 70 million people. Yet we are monitoring this vast protective system with almost no baseline health surveillance, a dangerous oversight as climate change threatens to dismantle it.

The stabilization mechanism is fragile: sparse vegetation (sustained by 110–300 mm annual rainfall) traps sand under low-wind conditions (drift potential <200 vector units) [3]. Climate projections for Central Asia indicate warming of up to 6 °C by 2100 under high-emission scenarios, with northward desert expansion already exceeding 100 km since the 1980s [4]. As precipitation declines and heat intensifies, vegetation loss triggers dune reactivation, transforming 500,000 km^2^ of stable substrate into an active dust source.

A severe dust storm in Uzbekistan in November 2021 generated PM_10_ concentrations exceeding 18,000 μg/m^3^, 260-fold above baseline, with PM_2.5_ elevated for nearly 10 days [5]. Chronic dust exposure in the region correlates with respiratory disease in children [6,7], and desert aerosols are increasingly linked to cardiovascular and infectious disease outcomes [8,9]. The health burden is predictable and quantifiable, yet no surveillance infrastructure exists to detect early destabilization signals or attribute disease burden to dust exposure.

The core problem is that a predictable crisis is approaching with no baseline to measure, prepare for, or prevent it. The UN Decade on Combating Sand and Dust Storms (2025–2034) provides the coordinating framework [10]. Central Asian health ministries and WHO regional offices must deploy air-quality networks and establish longitudinal cohort studies to link dust exposure to health outcomes. The United Nations Convention to Combat Desertification (UNCCD) and international consortia must embed health metrics into dust-monitoring programs and use satellite vegetation indices to track destabilization in real time. Every year of inaction narrows the window to build the baselines that will determine whether we can protect 70 million people as these sand seas shift from their current stability toward large-scale, climate-driven mobilization.

## CRediT authorship contribution statement

**Shivukumar Rakkasagi:** Writing – original draft, Conceptualization. **Dorit Nitzan:** Writing – review & editing, Supervision. **Shimrit Maman:** Writing – review & editing, Supervision, Funding acquisition, Conceptualization.

## Declaration of competing interest

The author declares that they have no competing interests.
